# Time-Resolved Visualisation of Nearly-Native Influenza A Virus Progeny Ribonucleoproteins and Their Individual Components in Live Infected Cells

**DOI:** 10.1371/journal.pone.0149986

**Published:** 2016-03-15

**Authors:** Sergiy Avilov, Julie Magnus, Stephen Cusack, Nadia Naffakh

**Affiliations:** 1 European Molecular Biology Laboratory, Grenoble Outstation, Grenoble, France; 2 University Grenoble Alpes-CNRS-EMBL International Unit (UMI 3265) for Virus Host-Cell Interactions, UMI 3265, Grenoble, France; 3 Institut Pasteur, Unité de Génétique Moléculaire des Virus à ARN, Département de Virologie, Paris, France; 4 CNRS, UMR 3569, Paris, France; 5 Université Paris Diderot, Sorbonne Paris Cité, Unité de Génétique Moléculaire des Virus à ARN, Paris, France; University of Rochester Medical Center, UNITED STATES

## Abstract

Influenza viruses are a global health concern because of the permanent threat of novel emerging strains potentially capable of causing pandemics. Viral ribonucleoproteins (vRNPs) containing genomic RNA segments, nucleoprotein oligomers, and the viral polymerase, play a central role in the viral replication cycle. Our knowledge about critical events such as vRNP assembly and interactions with other viral and cellular proteins is poor and could be substantially improved by time lapse imaging of the infected cells. However, such studies are limited by the difficulty to achieve live-cell compatible labeling of active vRNPs. Previously we designed the first unimpaired recombinant influenza WSN-PB2-GFP11 virus allowing fluorescent labeling of the PB2 subunit of the viral polymerase (Avilov et al., *J*.*Virol*. 2012). Here, we simultaneously labeled the viral PB2 protein using the above-mentioned strategy, and virus-encoded progeny RNPs through spontaneous incorporation of transiently expressed NP-mCherry fusion proteins during RNP assembly in live infected cells. This dual labeling enabled us to visualize progeny vRNPs throughout the infection cycle and to characterize independently the mobility, oligomerization status and interactions of vRNP components in the nuclei of live infected cells.

## Introduction

Influenza viruses are a global health concern because of the permanent threat of the emergence of novel strains potentially capable of causing pandemics [1, 2]. Moreover, seasonal influenza-related complications cause 250,000–500,000 deaths per year and considerable societal and economic cost. Thus, there is a need for a deeper understanding of the molecular mechanisms underlying the virus life cycle, which may lead to new therapeutic options to counter this unpredictable virus.

Influenza viruses are enveloped viruses with a genome consisting of 8 negative-sense single-stranded RNA segments. Each segment is a viral ribonucleoprotein (vRNP) complex composed of a pseudo-circular single strand RNA coated with multiple NP molecules; the heterotrimeric RNA-dependent RNA polymerase (composed of the PA, PB1 and PB2 subunits) is bound to the RNA duplex formed by the conserved complementary termini of the genomic RNA. Recent electron microscopy studies provided high resolution structures of native or recombinant influenza vRNPs [3, 4]. Replication and transcription of the viral genome occur in the nuclei of infected cells, being catalysed by the viral polymerase which uses vRNPs as templates [5, 6]. Thus correct assembly of the vRNPs is essential for the virus life cycle. NP oligomerization and NP-RNA interactions have been characterized *in vitro*, e.g. [7–10] and *in cellulo* [11–13]. It was reported that the NP residue D88 is involved in RNP activity and interaction with the PB2 polymerase subunit [14]. The interferon-inducible protein Mx1, which is well known to inhibit influenza virus replication, was found to interfere with the NP-PB2 interaction [15]. Whether the interaction between NP and PB2 is determinant for the host range of influenza A viruses is controversial [16–20]. The polymerase and NP have been shown to interact with many cellular proteins. An essential physical and functional interaction of the viral polymerase with the large fragment of the cellular RNA-dependent RNA polymerase II was described [21, 22]. A significant fraction of vRNPs is associated with the chromatin [23] and vRNP components interact with chromatin-associated factors such as PARP-1 [24] and HMGB1 [25]. Chromatin targeting of vRNPs in the same regions as Crm1 and Rcc1 could facilitate their export from the nuclei through the Crm1-dependent pathway [26]. There are many evidence that the Rab11 GTPase is involved in vRNP trafficking. It has been proposed that Rab11 mediates the docking of vRNPs to recycling endosomes which carry vRNPs towards the sites of viral assembly and budding at the plasma membrane (e.g., [27–29]). Despite these recent progress in the study of influenza vRNP assembly and trafficking, our knowledge on how these processes occur in live cell remains incomplete.

Direct observations of viral components in live infected cells by advanced fluorescence microscopy techniques can bring significant new insights into this field. To follow-up the time-dependent changes in composition and localization of viral proteins and vRNPs, as well as modifications of the cellular context which occur during the course of infection, we designed a recombinant influenza virus encoding a PB2 subunit that can be fluorescently labeled with a derivative of the GFP (Green Fluorescent Protein). To circumvent the fact that a virus expressing a PB2 subunit fused to the full length GFP could not be rescued, we adapted the split-GFP strategy [30, 31] to the virus. “Split-GFP” means that only a small fragment of the GFP (GFP11) is fused to a protein of interest, while the remaining part of the GFP (GFP1-10) is supplied independently within the cell and complements spontaneously with the GFP11 tag, yielding a GFP-like fluorophore called GFP_comp_. We developed a recombinant A/WSN/33 (H1N1) influenza A virus encoding the PB2 subunit of the polymerase fused to the GFP11 tag, further referred to as WSN-PB2-GFP11 [32, 33] (S1 Fig). PB2-GFP_comp_ was shown to be incorporated into the progeny vRNPs which were efficiently packaged into infectious virions. The WSN-PB2-GFP11 virus enabled us to visualize influenza polymerase in live cells throughout the infection cycle [32, 33]. More recently, Lakdawala et al. used an influenza virus encoding a PA polymerase subunit tagged with the full length GFP to track vRNPs in the cytoplasm of live cells [34].

**Fig 1:**
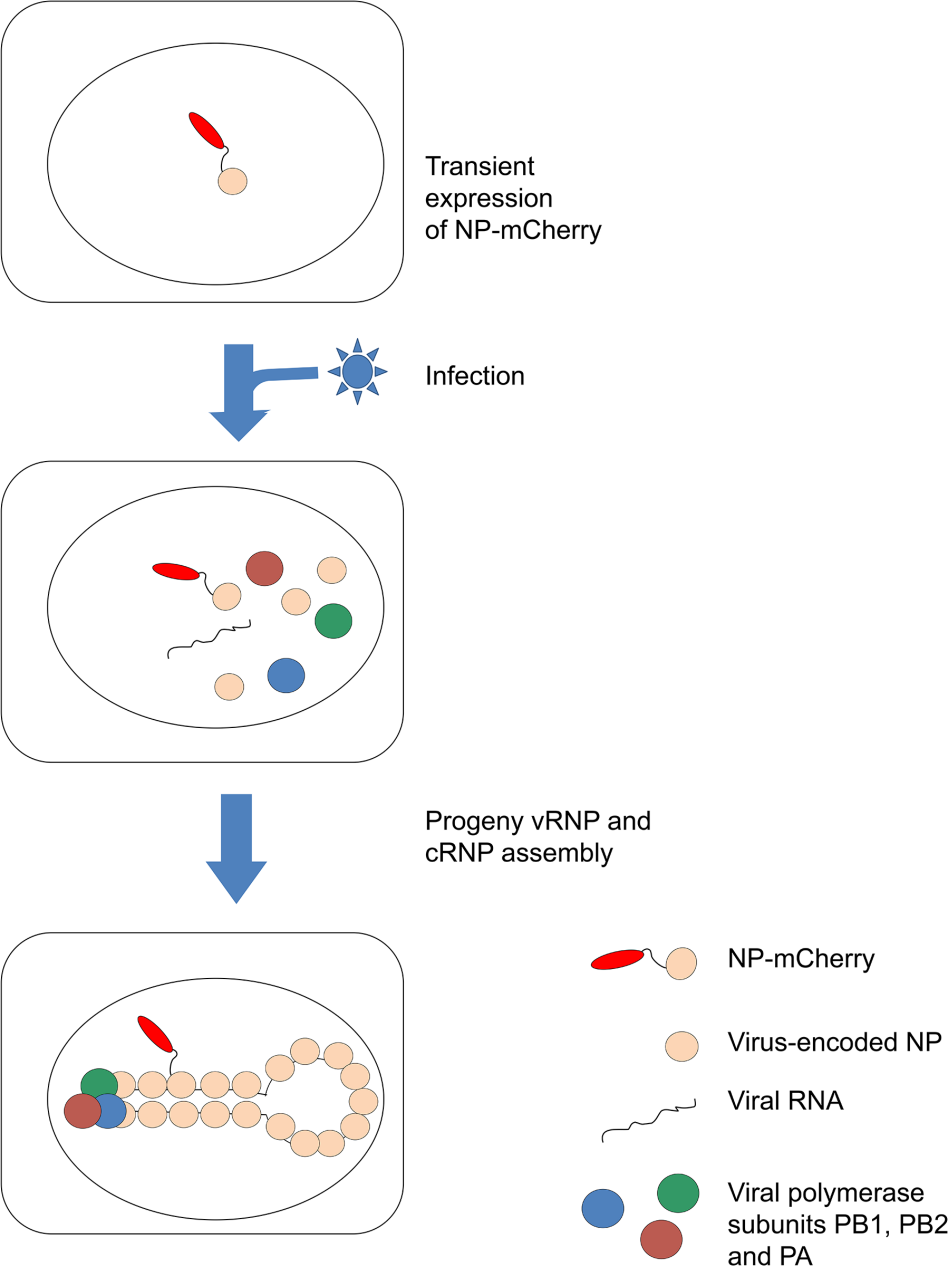
Principle of the labeling of influenza vRNPs with transiently expressed NP-mCherry proteins. Image is not to scale. Neither the real stoechiometry nor the spatial organization of a vRNP is represented. For clarity, only a single NP-mCherry incorporated into an RNP complex is shown, although incorporation of multiple NP-mCherry molecules per RNP might also occur.

However, labeling of the viral polymerase is not optimal to study certain steps of the influenza virus life cycle. For instance, it is not suitable for tracking the progeny vRNPs in the nuclei, because a subpopulation of free polymerases is likely present in that compartment. Fluorescent labeling of vRNPs themselves is needed, preferably in combination with labeling of the polymerase. Transfection of a fluorescently labeled specific antibody was used to characterize instantaneous movements of vRNPs in the cytoplasm of infected cells with short observation time periods [28]. However, in order to observe vRNPs both in the cytoplasm and in the nucleus over extended periods of time and to avoid problems inherent to live-cell immunostaining, labeling of vRNPs through a genetically encoded tag is needed. As vRNPs reconstituted in a transient expression system with fluorescently-tagged NPs show no detectable transcription/replication activity (N. Naffakh, unpublished), possibly due to a defect in NP oligomerization and/or NP-RNA interaction, we reasoned that vRNPs containing only a few labeled NP molecules would be more likely to retain their activity. Here we combined labeling of the viral polymerase PB2 subunit through GFP1-10/GFP11 complementation, and labeling of vRNPs through spontaneous incorporation of transiently expressed NP-mCherry proteins (Fig 1). The latter did not require any additional genetic modification of the WSN-PB2-GFP11 virus and did not perturb viral replication. The labeling approach allowed us to characterize independently and simultaneously the mobility and oligomerization status of the NP and viral PB2 protein as well as their interactions in the nuclei of live infected cells, using fluorescence correlation spectroscopy (FCS) and Forster Resonance Energy Transfer (FRET) techniques.

## Materials and Methods

### Cells, Viruses and Plasmids

pCMV-GFP1-10 and pCMV-GFP11 plasmids were purchased from Sandia Biotech (USA). MBD-GFP11 (maltose binding domain-GFP11) and mCherry-GFP11 constructs were described before [33, 35–37]. In the Cherry-GFP11 construct, the mCherry and GFP11 tag are connected by a 17-amino acid linker. The untagged recombinant influenza A/WSN/33 (further referred to as WSN-wt) and WSN-PB2-GFP11 viruses were produced by reverse genetics and amplified on MDCK cells as described in [33]. The A549-GFP1-10 cell line stably expressing the GFP1-10 fragment was obtained upon lentiviral transduction, as described in [33]. HEK-293T, Vero and A549-GFP1-10 cells were grown in complete Dulbecco's modified Eagle's medium (DMEM) without phenol red, supplemented with 10% fetal bovine serum (FBS), glutamine, sodium pyruvate, penicillin and streptomycin.

### Infection and Virus Growth Assay

For imaging experiments sub-confluent HEK-293T, A549-GFP1-10 or Vero cells were plated on LabTekII chambered cover glasses (Nunc), 8-well Ibidi μ-Slides (Ibidi GmbH, Germany), or on glass coverslips (for immunofluorescence and PLA assays, see below), and transfected with the appropriate plasmid(s) using the Fugene-HD or Fugene 6 transfection reagents (Roche). At 24 or 48 hours post-transfection (hpt), the cells were incubated for 1 h at 37°C with the WSN-wt or the WSN-PB2-GFP11 virus at a multiplicity of infection (MOI) of 3–5 PFU/cell (plaque forming units per cell) in DMEM, or mock-infected. After virus adsorption, the inoculum was replaced by the complete DMEM medium described above.

Knock-down of Rab11 was achieved by transfecting commercial validated siRNAs directed to the human Rab11A and Rab11B proteins or scrambled non-target siRNAs as a negative control (Life technologies) into adherent A549 cells on LabTekII chambered cover glasses. Three days after transfection, the cells were infected at a high MOI, as described above.

For virus growth assays, confluent monolayers of HEK-293T cells plated in 96-well plates were transfected with 200 ng of the indicated plasmids and infected at 24 hpt with the WSN-wt virus at a MOI of 0.001 PFU/cell. Following 1 h of adsorption at 35°C, cells were incubated in DMEM supplemented with 2% FBS for 48 hours. The supernatants were harvested and titrated by a plaque assay on MDCK cells as described previously [38].

### Fluorescence Microscopy

For live cell fluorescence microscopy, the cells were plated on LabTekII chambered cover glasses (Nunc, USA) or on 8-well Ibidi μ-Slides (Ibidi, Germany) and kept at 37°C in 5% CO_2_ atmosphere during image acquisition. Images except time-lapse series were acquired on confocal laser scanning microscopes: SP2 AOBS (acousto-optic beam splitter) (Leica Microsystems, Germany) and LSM780 (Carl Zeiss, Germany), both with 63x 1.4 N.A. oil immersion objectives. Time-lapse series were acquired on Andor Revolution Nipkow spinning disk microscope equipped with DV885 EMCCD camera (Andor Technologies, USA) and CSU22 confocal scan head (Yokogawa, Japan), with a 63x water immersion objective. On the scanning confocal microscopes, excitation and emission were set at 488 and 500–550 nm for GFP and FITC (fluorescein isothiocyanate), 405 and 420–470 nm for DAPI (4',6-diamidino-2-phenylindole), 561 and 590–670 nm for mCherry, 633 nm and 643–742 nm for AlexaFluor647. On the Nipkow disk microscope, standard dichroic and filter combinations for GFP and RFP (red fluorescent protein) fluorescence were used to acquire GFP_comp_ (the fluorophore formed by complementation of non-fluorescent GFP1-10 and GFP11 fragments) and mCherry signals with excitation by 488 nm and 561 nm lasers, respectively. In all multicolor imaging, signals were acquired sequentially. Co-localization analysis was performed with ImageJ 1.44c program (http://rsb.info.nih.gov/ij/); Pearson correlation coefficient was calculated; occurrence of false co-localization was excluded by calculating Pearson coefficient for “scrambled” images according to the Costes method using the *Co-localization Test* plug-in of ImageJ. Images were corrected for possible cross-talk between the channels; for presentation purpose, brightness and contrast of raw images were adjusted.

### Indirect Immunofluorescence Assay

Infected or mock-infected HEK-293T, A549 or Vero cells were fixed with PBS-4% paraformaldehyde (PFA) for 20 min, permeabilised with PBS-0.1% Triton X100 for 10 min, treated with 3% normal rabbit serum for 60 min, and then incubated with the anti-influenza virus NP monoclonal antibody, clone 3/1 [27], further referred as “anti-NP antibody 3/1” (a kind gift of Prof. Robert G. Webster, St. Jude Children’s Research Hospital, Memphis, TN, USA) diluted 1: 200. Subsequently, cells were incubated with AlexaFluor647-coupled or FITC-coupled anti-mouse secondary antibody diluted 1: 500 (Molecular Probes and Santa-Cruz Biotechnology) and stained with DAPI.

### Proximity Ligation Assay (PLA)

HEK-293T cells cultured on poly-lysine coated glass coverslips in 6-well plates were transfected with 400ng of the NP-mCherry or mCherry-NLS (nuclear localization signal) plasmid using the Fugene6 reagent (Roche), next day infected with the WSN-wt virus or mock-infected, fixed and stained with a mixture of the mouse anti-NP antibody 3/1 diluted 1: 100, and the rabbit Living Colors^®^ DsRed Polyclonal Antibody (which recognizes mCherry as well, Clontech, Cat. #632496) diluted 1: 100, as described above. Further, to detect *in situ* if the epitopes are within 40 nm proximity [39], cells were subjected to proximity ligation assay with DuoLink II fluorescence kit (Sigma) according to the manufacturer’s protocol. Anti-mouse PLA probe MINUS, anti-rabbit PLA probe PLUS and Detection Reagents Far Red were used. PLA signal, mCherry fluorescence and DAPI signals were acquired with excitation at 633 nm, 561 nm, and 405 nm, respectively, and standard emission wavelength ranges for far red, red and blue color channels. Experimental infected samples were compared to the samples with omitted primary antibodies and with mock-infected samples. Stacks of optical slices were acquired on laser scanning confocal microscope. For quantification, the PLA “spots” were automatically detected in the 3D image stacks and counted with Measurements module of Imaris 8.1.2 software (Bitplane, Switzerland).

### FCS

Fluorescence correlation spectroscopy (FCS) measurements were performed with Confocor 3 system built on Zeiss AxioObserver Z1 inverted microscope equipped with 40x 1.3NA water immersion objective and avalanche photodiode detectors (Carl Zeiss, Germany), as described in detail before [40]. 488 nm and 561 nm diode lasers were used to excite GFP and mCherry fluorescence; the emission was separated with standard sets of filter cubes. The fluorescence fluctuation data were hardware-correlated, and the correlation curves were subsequently fitted with Zen software (Zeiss) to the single-component or two-component translational diffusion models.

### FLIM-FRET

Fluorescence lifetime imaging (FLIM) using two-photon excitation and time-correlated single photon counting was performed on Zeiss LSM 510 NLO META FLIM system (Carl Zeiss, Germany) equipped with a Tsunami Ti:Sapphire pulsed laser (Spectra Physics, USA) and on Zeiss LSM 710 NLO system equipped with a Cameleon, Vision II Ti:Sapphire pulsed laser, both with 40x oil immersion objectives. The two-photon excitation pulses at repetition rate of 80 MHz were tuned to 950 nm. The emission of GFP_comp_ was collected at 505–550 nm. The single photon events were detected with a single photon counting photomultiplier H7422 (Hamamatsu, Japan) (LSM 510 NLO META FLIM) or GaAsP-APD hybride “photon counting” detector (LSM 710 NLO), and recorded on a SPC830 analyzer (Becker&Hickl, Germany); time-resolved fluorescence data were handled and analyzed by *SPCImage* software (Becker&Hickl, Germany). Fluorescence intensity decays were deconvolved with the measured instrument response function and analysed as a monoexponential:

$$I\left(t\right)\;=\;I_{0}exp\left(-t/\tau \right)$$

where *I(t)* is the fluorescence intensity at time *t*. FRET efficiency (*E*) was calculated as *E* = 1−*τ*_*DA*_*/τ*_*D*_ (1), where *τ*_*DA*_ and *τ*_*D*_—mean fluorescent lifetime in the presence and in the absence of the acceptor, respectively [41]. Mean lifetime measured in negative control samples (co-expressed non-interacting MBD-GFPcomp and mCherry) was used as *τ*_*D*_.

## Results

### Transiently Expressed NP-mCherry Co-Localizes with vRNPs in Influenza Virus-Infected Cells

**Fig 2:**
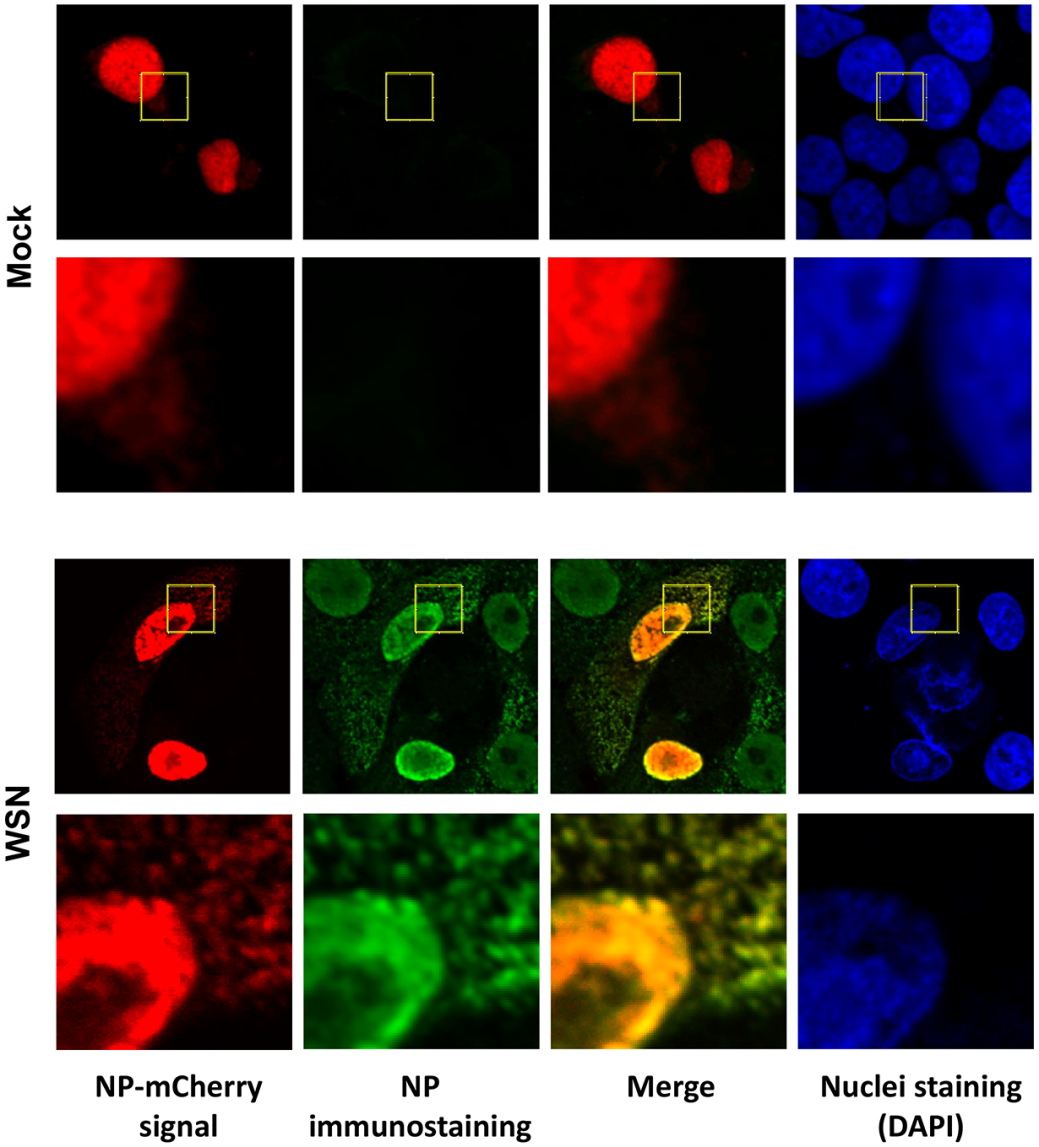
Colocalization of transiently expressed NP-mCherry with vRNPs in infected A549 cells. Mock-infected cells (top panels) or cells infected with the WSN-wt influenza virus (bottom panels) are shown. Cells were fixed at 6 hpi and stained with the anti-NP monoclonal antibody clone 3/1. Scale bar: 10 μm. Pseudocolors: red, mCherry; green, NP; blue, nuclei staining (DAPI).

We sought to label influenza vRNPs in infected cells by incorporation of “heterologous” NP-mCherry fusion proteins during their assembly (Fig 1), in order to characterize their localization, mobility and molecular interactions in live cells during infection. HEK-293T cells were transfected with a plasmid encoding NP-mCherry. The day following transfection, cells were mock-infected or infected with the WSN-wt virus at a high MOI, fixed, and immunostained with the anti-NP antibody 3/1 reported to specifically recognize vRNPs in an infectious context [27] (see below). In mock-infected cells, NP-mCherry auto-fluorescence was mostly nuclear and only a weak signal was visible in the cytoplasm without any punctuate pattern (Fig 2, top left panel), in agreement with the commonly observed pattern of individually expressed NP (e.g. [42]). The NP immunostaining signal in mock-infected cells was very low in both the nucleus and the cytoplasm (Fig 2), indicating that the anti-NP antibody 3/1 does not recognize the NP-mCherry protein when expressed alone. In infected cells, NP-mCherry appeared not only in the nuclei, but also as puncta in the cytoplasm, with a high degree of co-localization with the NP immunostaining (Fig 2, bottom panels), as confirmed by co-localization analysis: the Pearson coefficient was 0.68±0.20 for the nuclei and 0.56±0.15 for the cytoplasm at 6–7 hours post-infection. This suggests that at least a sub-population of NP-mCherry is incorporated into vRNPs, as neither free NP nor cRNPs accumulate in cytoplasmic puncta-like entities thought to be associated to recycling endosomes [27–29].

To obtain another hint about the specificity of the anti-NP antibody 3/1 for vRNPs [27], we observed its staining pattern in infected cells upon knock-down of the Rab11 protein (S2 Fig). Rab11 is known to contribute to the trafficking of influenza vRNPs towards the plasma membrane. In control cells, NP staining showed the typical punctuate pattern commonly observed for NP at late times post-infection. Upon Rab11 knock-down the cytoplasmic NP staining became diffuse. Interestingly, this NP immunostaining pattern was very similar to that observed in cells expressing a dominant negative mutant of Rab11 (Fig 4A in [28]). This dependence of the staining pattern on Rab11 suggests that the anti-NP antibody 3/1 indeed recognizes mostly vRNPs, because neither cRNPs nor free NP are subject to Rab11-dependent vesicular trafficking.

### PLA Assay Indicates Proximity between mCherry and vRNPs in Influenza Virus Infected Cells Transiently Expressing NP-mCherry

Although the localization pattern of NP-mCherry and its co-localization with NP-positive puncta in the cytoplasm of immunostained infected cells suggest that NP-mCherry is incorporated into vRNPs, they do not prove it. To detect close interactions between NP-mCherry and vRNPs and to gain information on the sub-cellular localization of the complexes, we performed proximity ligation assay (PLA). In PLA, a signal is generated only when two oligonucleotide-labeled secondary antibodies (“PLA probe PLUS” and “PLA probe MINUS”) are bound in proximity (<40 nm). As a result of ligation which yields a closed DNA circle, followed by rolling-circle amplification by a DNA-dependent DNA polymerase and hybridization of the amplification product with fluorescently labeled oligonucleotides, each pair of PLA probes is visualized as a single fluorescent spot. Then, the number of complexes is estimated by counting these fluorescent spots [39].

**Fig 3:**
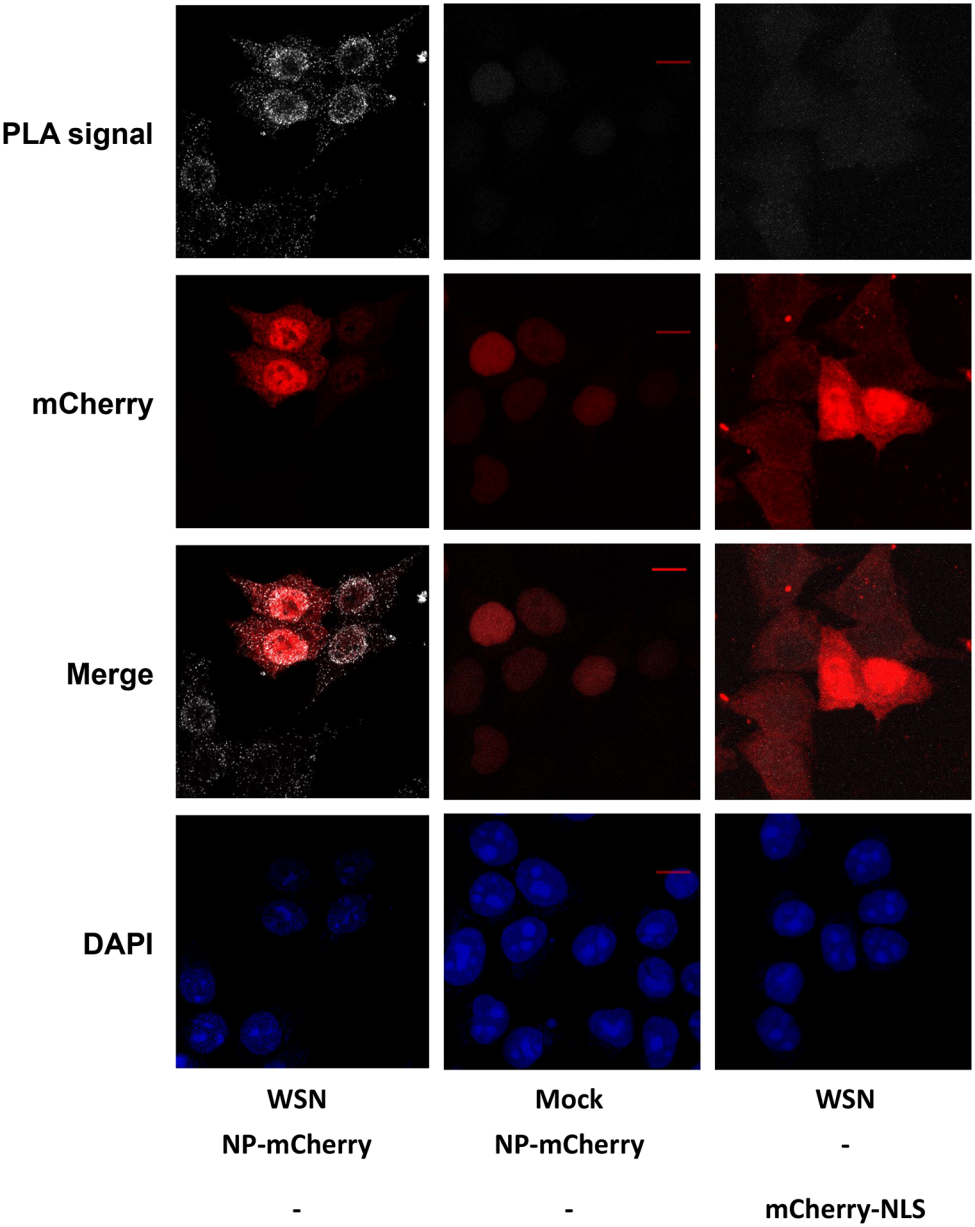
Detection of proximity between NP-mCherry and vRNPs in infected cells by proximity ligation assay. The anti-NP monoclonal antibody 3/1 and a rabbit antibody recognizing mCherry were used. Maximal intensity projections of the z-stacks acquired with a laser scanning confocal microscope are shown. Scale bar: 10 μm. Pseudocolors: white, PLA signal; red, mCherry; blue, nuclei staining (DAPI).

Here, WSN-infected HEK-293T cells were incubated with a mixture of the mouse anti-NP 3/1 antibody and the rabbit antibody against mCherry (details in Materials and Methods) and next incubated with a mixture of the “mouse PLA probe MINUS” and “rabbit PLA probe PLUS”. In the nuclei of infected cells fixed at 4 hpi, 147±106 spots/cell were observed (representative image on Fig 3, left column). In the mock-infected cells transfected with NP-mCherry, PLA signal was substantially weaker and much less PLA spots were detected by the software (14±10 spots/cell) (Fig 3, middle column). In the second control, i.e. cells transfected with an mCherry-NLS construct instead of NP-mCherry and infected with the WSN-wt virus (Fig 3, right column), the mCherry-NLS signal had the same intensity as the NP-mCherry signal (Fig 3, left column) but zero to 2 PLA spots per cell were detected. Overall, taken together with the fact that in our transfection and immunostaining conditions the anti-NP antibody 3/1 does not bind significantly to transiently expressed NP-mCherry and recognizes mostly vRNPs (Fig 2 and S2 Fig), the PLA assay showed that in the nuclei of infected cells, at least some proportion of the transiently expressed NP-mCherry and the virus-encoded RNPs are in close proximity (< 40 nm). Some NP-mCherry molecules are thus likely incorporated into vRNPs.

### NP-mCherry Re-Localizes from the Nucleus to the Plasma Membrane during the Course of Infection Simultaneously with PB2-GFP_comp_

**Fig 4:**
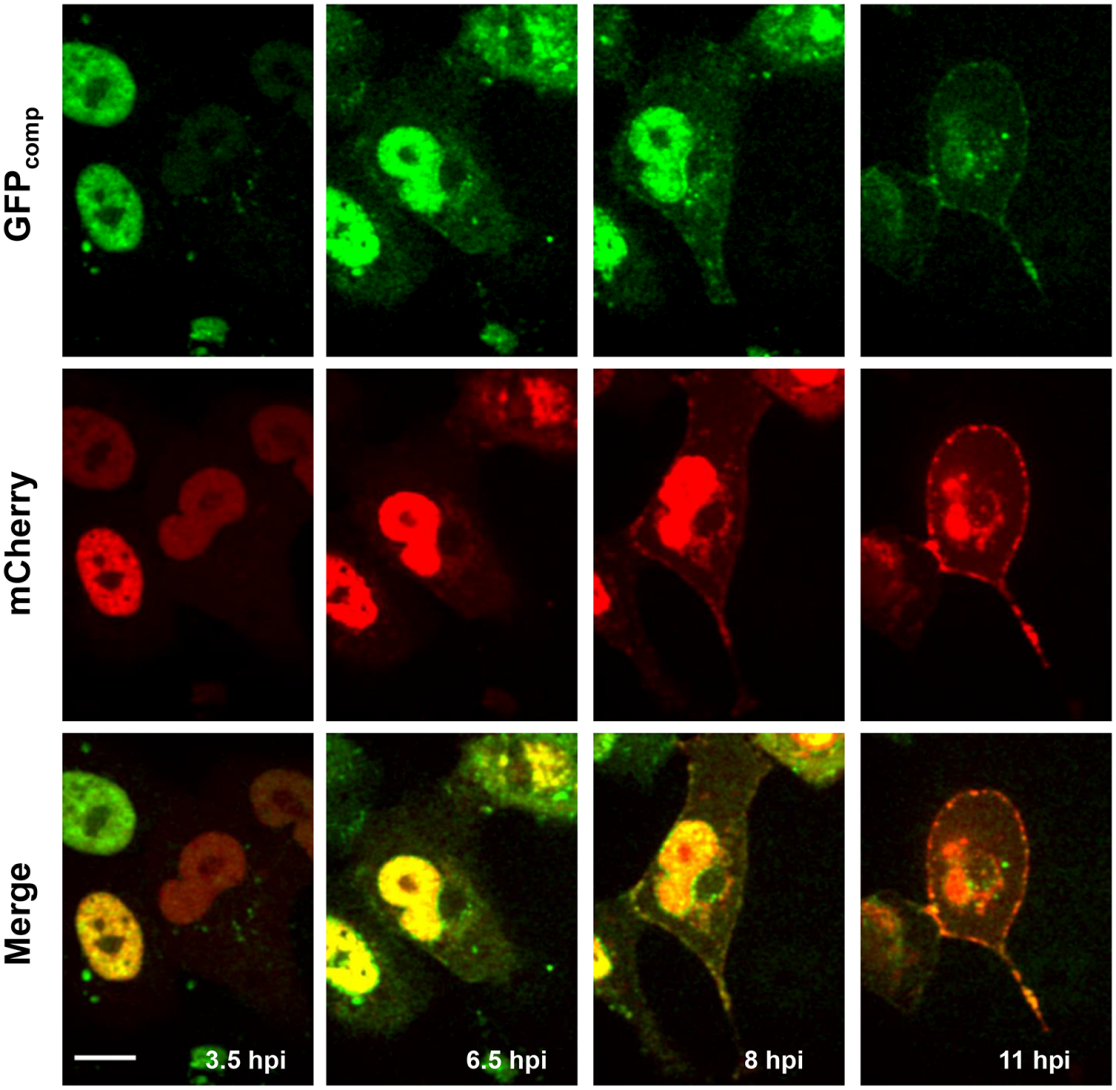
Live Vero cells transfected with GFP1-10 and NP-mCherry and infected with the WSN-PB2-GFP11 virus. Selected frames from S1 Movie at the indicated times post-infection are shown. Pseudocolors: green, PB2-GFP_comp_; red, NP-mCherry. Scale bar, 10 μm. Time-lapse series of single optical slices were acquired with a Nipkow spinning disk microscope.

To characterize the localization of transiently expressed NP-mCherry and virus-encoded PB2 throughout the infection cycle, the mCherry and GFP_comp_ signals were continuously acquired over extended periods of time in individual Vero cells after infection with the WSN-PB2-GFP11 virus (Fig 4 and S1 Movie). Early after infection, NP-mCherry and PB2-GFP_comp_ were detectable only in the nucleus (Fig 4, left column). At 6.5 and 8 hpi, NP-mCherry appeared in the cytoplasm (Fig 4, middle columns). At later time points it accumulated at the plasma membrane, in agreement with known accumulation of progeny vRNPs at the sites of virion assembly [43], and showed strong co-localization with PB2-GFP_comp_ at the plasma membrane (Fig 4, right column and S1 Movie). This behavior of NP-mCherry during the course of infection was very distinct from its steady-state nuclear accumulation in the nuclei in non-infected cells (data not shown). The most likely explanation is that NP-mCherry is incorporated into progeny vRNPs and that mCherry-labeled vRNPs move towards the plasma membrane. Thus, our time-lapse observations are in agreement with the immunofluorescence co-localisation and PLA data described above (Figs 2, 3 and S2), and indicate an incorporation of NP-mCherry into progeny vRNPs.

### NP-mCherry Does Not Interfere with Influenza Virus Multi-Cycle Replication

Fluorescent protein tags are large and may disrupt normal functions of the labeled entities. Thus we evaluated whether transiently expressed NP-mCherry protein could interfere with the normal process of viral replication. HEK-293T cells were transiently transfected with NP-mCherry, the mutant NP_R416A-mCherry, an mCherry protein fused to a nuclear localization signal (mCherry-NLS) or a control empty plasmid. The NP_R416A mutant, in which the domain exchange loop (residues 402–421) cannot interact with another NP protomer, remains monomeric and has a significantly lower affinity to RNA [44, 45]. It was included in our experiments to investigate whether NP-PB2 interactions could occur independently of NP oligomerization and NP binding to RNA (see below). Transfected cells were infected 24 h later with the WSN-wt virus at a low MOI of 0.001 PFU/cell. NP-mCherry and NP_R416A-mCherry were expressed in more than 95% of the cells at the time of infection, as assessed by fluorescence microscopy (S3 Fig). Supernatants were harvested at 48 hours post-infection and titrated by plaque assay on MDCK cells. Viral titers were in the same range in the presence or in the absence of NP-mCherry and NP_R416A-mCherry constructs (Table 1). Thus, transiently expressed NP-mCherry and NP_R416A-mCherry do not inhibit multi-cycle replication of influenza virus in HEK-293T cells.

**Table 1:** Effect of transient expression of NP-mCherry constructs on the production of infectious viral particles.

Transfected construct	Virus titer (PFU/ml)
**NP-mCherry**	4.0×10^4^
**NP_R416A-mCherry**	2.0×10^4^
**NP**	3.2×10^4^
**mCherry-NLS**	3.0×10^4^
**Empty vector**	4.5×10^4^

### Fluorescence Correlation Spectroscopy Study of PB2-GFP_comp_ and NP-mCherry- Containing Species in the Nuclei of Live Infected Cells

**Fig 5:**
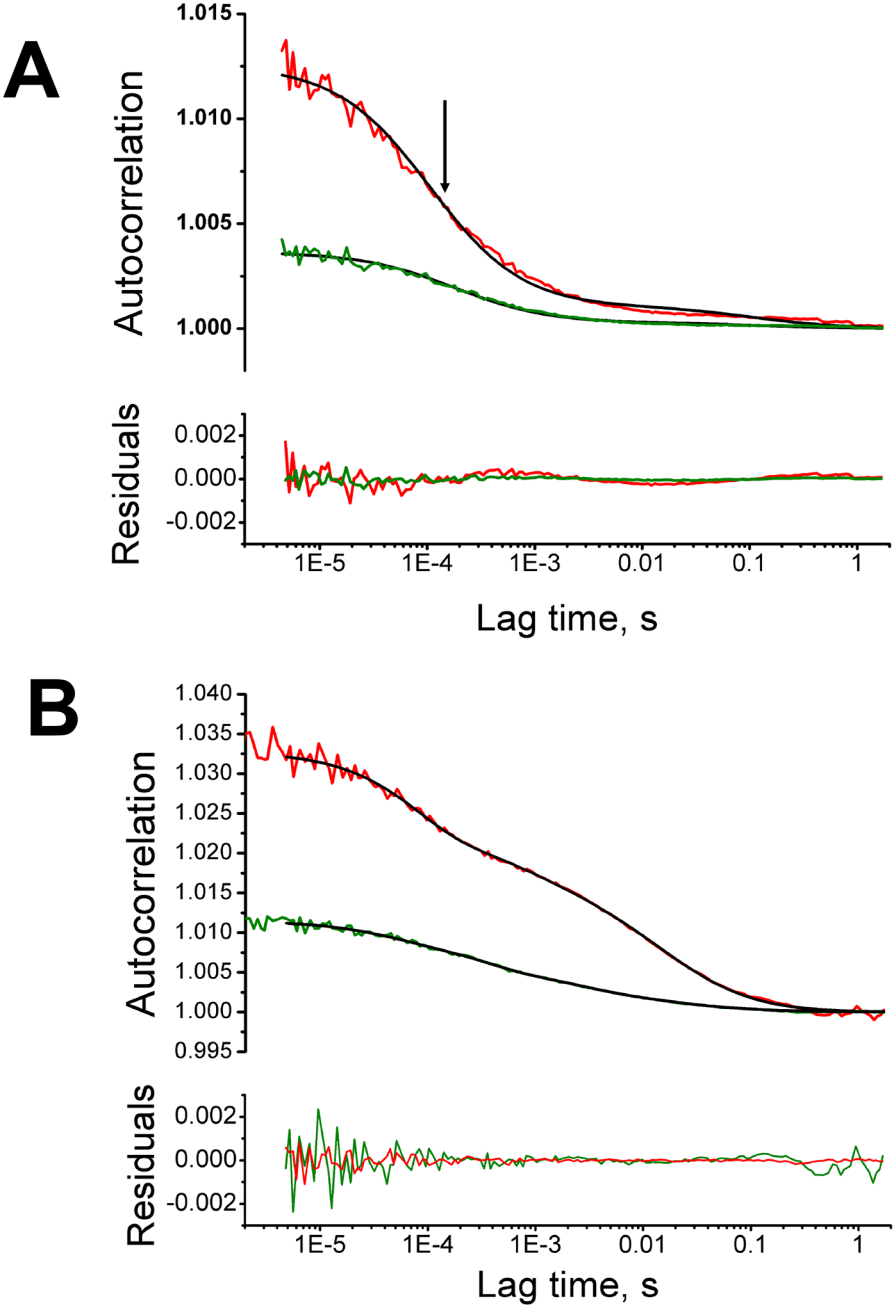
Fluorescence correlation spectroscopy data for GFP_comp_-labeled species (green) and mCherry-labeled species (red). The experimental autocorrelation data, fitted curves (in black), and residuals, are shown for an individual representative HEK-293T cell. A. Control cell transiently expressing MBD-GFP_comp_ and mCherry and infected with the WSN-wt virus; single-component translational diffusion model. Arrow points to the inflection point of the autocorrelation curve. B. Cell transfected with GFP1-10 and NP-mCherry and infected with the WSN-PB2-GFP11 virus. Two-component translational diffusion model.

Fluorescence correlation spectroscopy (FCS) is a well-established technique enabling to probe mobility, concentration and oligomerization of fluorescently labeled species. It is based on a sophisticated analysis of fluorescence signal fluctuations in a small (femtoliter range) detection volume [46, 47]. FCS is usually performed on a confocal microscope setup and is compatible with live cell samples. For instance, FCS allows to determine the translational diffusion time, which corresponds to the inflection point on the sigmoidal graph of the fluorescent signal autocorrelation function *versus* time (indicated by an arrow in Fig 5A). FCS also provides an intensity-independent estimate on the number of labeled particles in the FCS detection volume. From this, one can estimate brightness/particle ratio, which serves as a measure of the oligomerization status of the labeled species.

Here, to obtain information on the mobility and the oligomerization status of NP- and PB2-containing species in the nuclei of infected cells, we acquired FCS data on HEK-293T cells transiently expressing GFP1-10. Two types of samples were characterized: 1) infected cells containing virus-encoded PB2-GFP11 and transiently expressing NP-mCherry, in which presumably most PB2 molecules were labeled by GFP_comp_, a small proportion of NP was labeled with mCherry, and all other components expressed by the virus were unlabeled; 2) mock-infected cells transiently expressing NP-mCherry and PB2-GFP11, mostly converted in PB2-GFP_comp_, in the absence of any other viral protein or RNA. To obtain a negative control containing non-interacting and non-oligomerizing green and red fluorescent species, HEK-293T cells were co-transfected with mCherry, GFP1-10 and MBD-GFP11 (Maltose-Binding-Domain fused to GFP11). To exclude non-specific effects of infection on the FCS results (e.g., due to a putative change in microviscosity), negative control samples were infected at a high MOI with the untagged WSN-wt virus. No difference with the non-infected negative control was observed (data not shown). Fitting of FCS autocorrelation curves for both mCherry and MBD-GFP_comp_ species in the negative control yielded a single translational diffusion time of 0.2–0.4 ms (Fig 5A and Table 2, sample #1), in line with the values commonly observed for monomeric non-interacting proteins in a cell [12, 47, 48]. When PB2-GFP_comp_ and NP-mCherry were co-expressed, good fitting of autocorrelation curves required a model with two translational diffusion times, in non-infected cells (Table 2, sample #2) as well as in infected cells (Fig 5B and Table 2, sample #3). This indicated the presence of at least two species possessing distinct mobilities. The shorter, sub-millisecond diffusion time was close to the value observed for the negative control (Table 2, sample #1), and thus represented subpopulations of PB2-GFP_comp_ and NP-mCherry which neither formed large oligomers nor interacted with any “slow” cellular or viral partners such as RNA. The longer diffusion time, in the range of 5–15 ms, obviously represented much slower moving species. In general, these “slow” species might occur due either to oligomerization or to interaction with immobile or slowly moving structures. Translational diffusion time of a particle is proportional to the cubic root of its hydrodynamic radius (and to molecular mass in the first approximation), therefore a 10-fold increase of diffusion time would correspond to a 1000-fold increase of molecular mass. However, molecular brightness did neither increase drastically for PB2-GFP_comp_ nor for NP-mCherry fluorophore (Table 2), which indicates that no large-scale homo-oligomerization occurred. Therefore, the PB2-GFP_comp_ and NP-mCherry-containing species with longer diffusion time are associated with slowly moving or motionless structures which might belong to the chromatin environment [23, 26].

**Table 2:** Fluorescence correlation spectroscopy data for PB2-GFP_comp_- and NP-mCherry-labeled species in the nuclei of HEK-293T cells.

Sample #*	mCherry signal					GFP_comp_ signal				
	Translational diffusion time 1, ms	Component 1 fraction, %	Translational diffusion time 2, ms	Component 2 fraction, %	Molecular brightness	Translational diffusion time 1, ms	Component 1 fraction, %	Translational diffusion time 2, ms	Component 2 fraction, %	Molecular brightness
**#1: MBD-GFP11 + mCherry + WSN virus**	0.16±0.04	100	-	0	1.0±0.5	0.41±0.11	100	-	0	1.0±0.10
**#2: PB2-GFP11+ NP-mCherry + Mock-infection**	0.09±0.003	72±2.2	6.9±0.7	28±2.2	1.2±0.2	0.18±0.03	69±2.1	4.9±1.1	31±2.1	0.7±0.52
**#3: NP-mCherry + WSN-PB2-GFP11 virus**	0.11±0.01	48±6.8	15±2.7	52±6.8	1.6±0.3	0.22±0.06	68±3.8	11±4.8	32±3.8	1.1±0.16
**#4: NP_R416A-mCherry + WSN-PB2-GFP11 virus**	0.08±0.02	68±2.0	5.1±1.5	32±2.0	0.8±0.2	0.26±0.03	69±3.4	13±3.5	31±3.4	1.1±0.10

* GFP1-10 was transiently expressed in all samples, in addition to the plasmids mentioned in the first column; values ± SD are shown. Data were acquired and analyzed as described in *Materials and Methods*.

The infected cells differed from the mock-infected cells as the fractions of “slow” species of NP-mCherry in the nucleus represented about 50% and 30%, respectively; moreover, the corresponding effective diffusion time increased to 15 ms, as compared to 7 ms in the absence of infection (Table 2, sample #3 compared to sample #2). Therefore, besides the subpopulation of NP interacting with cellular components observed in non-infected cells, additional slowly diffusing species occurred in the infected cells. A likely explanation is that they represent vRNPs containing at least one NP-mCherry molecule, as detected in our co-localization and PLA assays described above. This hypothesis is strengthened by the fact that, in contrast to the wild-type NP-mCherry, the NP-mCherry_R416A oligomerization-impaired mutant showed the same FCS parameters in infected and non-infected cells (Table 2, sample #4).

For PB2-GFP_comp_, the fraction of the “slow” species remained roughly the same in non-infected and infected cells (about 30%), but the translational diffusion time of the “slow” species was twice longer in infected cells compared to non-infected cells expressing PB2-GFP_comp_ (11 ms and 5 ms respectively) (Table 2, sample #3 compared to sample #2). Therefore, in the context of infection, a subpopulation of PB2-GFP_comp_ was incorporated into a species which diffuses much slower than the PB2-GFP_comp_-containing complexes in the absence of infection. The RNP-bound PB2-GFP_comp_ likely constitute a fraction of “slow” species in the infected cells, considering the data reported here (Fig 4 and S1 Movie) and our previous results [32] showing that after initial accumulation in the nuclei, PB2-GFP_comp_ is exported to the cytoplasm in a CRM1-dependent manner, accumulates in recycling endosome and is finally concentrated at the plasma membrane [32, 33]. However, we cannot exclude that hetero-trimeric viral polymerase also contribute to the “slow” diffusing time observed for PB2-GFP_comp_, as we previously observed slowly diffusing species in uninfected cells transiently co-expressing PB2 and the two other subunits of the polymerase, PB1 and PA [40]. The remaining fraction of 70% of PB2-GFP_comp_ in the infected cells might correspond to PB2-GFP_comp_ molecules that undergo rapid binding/dissociation to vRNPs, or might represent an excess of PB2-GFP_comp_ molecules unbound to vRNPs. In line with the latter hypothesis, we systematically observed a substantial amount of PB2-GFP_comp_ in the nucleus at late times post-infection (8–11 hpi), when vRNPs were largely exported out from the nucleus (S1 Movie).

### PB2-GFP_comp_−NP-mCherry Interactions in the Nuclei of Live Infected Cells

Although PLA signal indicates proximity (<40 nm) between the two antigens [39], it does not necessary prove a direct interaction. To investigate whether direct interactions of PB2 with NP and/or vRNP occur in live infected cells, we used FRET microscopy, which is one of few well-established techniques for the visualization of molecular interactions in living cells. FRET phenomenon is non-radiative transfer of the energy of an excited fluorophore (the donor) to another fluorophore (the acceptor), and enables one to detect the fluorescently labeled molecules separated by less than 5–10 nm. Considering sizes of fluorescent protein tags and typical globular proteins, the fluorophores can be brought to such proximity by direct interactions between the tagged proteins (or between tags themselves). Furthermore, FRET may only occur during the “life” of a fluorophore in excited state, which is few nanoseconds for GFP; therefore, “false-positive” FRET due to accidental collisions between non-interacting fluorescent protein donor and acceptor is very unlikely. Fluorescence lifetime microscopy (FLIM) detects FRET as a decrease of the donor fluorescence lifetime in the presence of the acceptor. In contrast to fluorescence intensity, the fluorescence lifetime does not depend on label concentration and sample geometry, which makes lifetime measurement the most reliable way to detect FRET.

**Fig 6:**
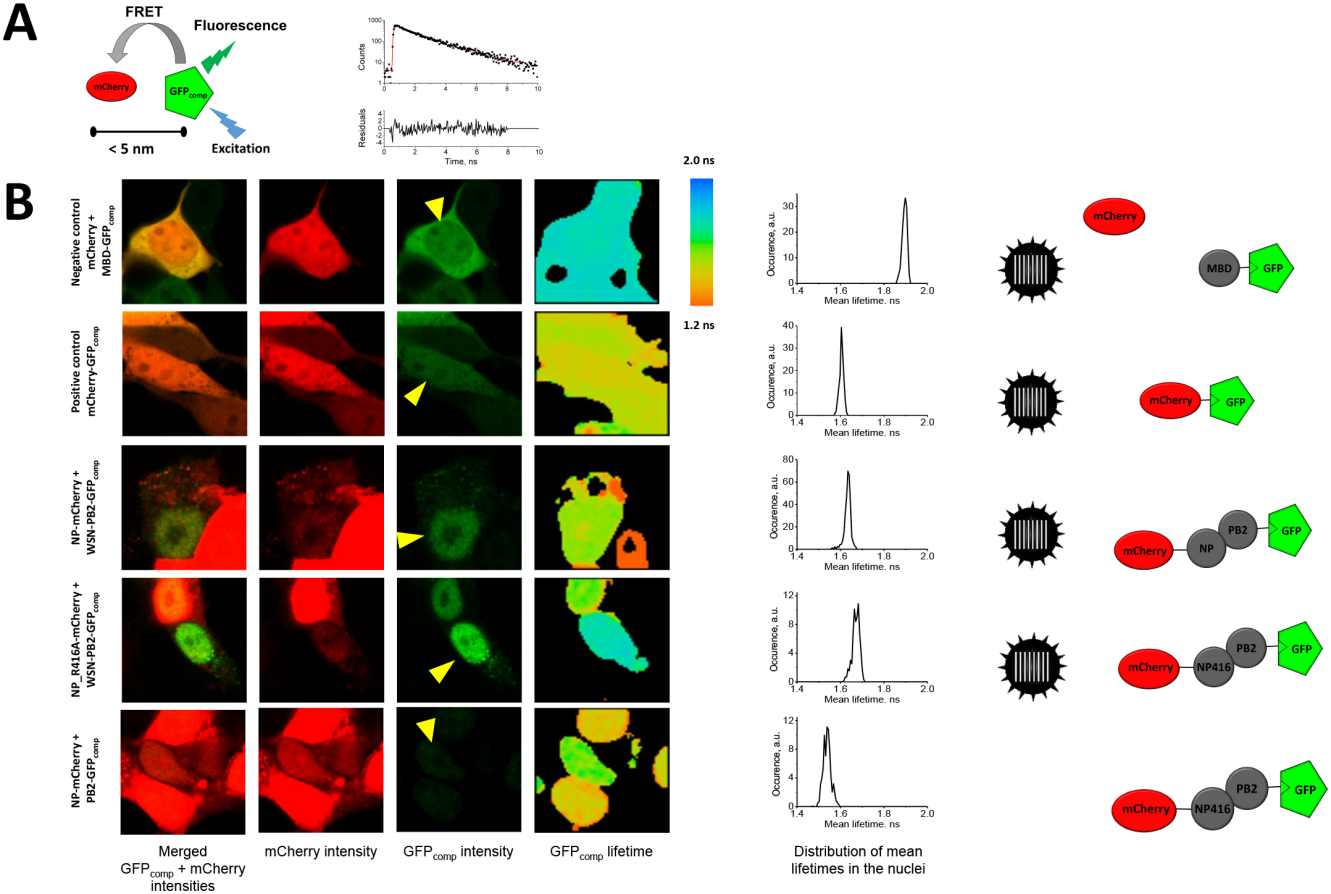
FLIM-FRET microscopy in live HEK-293T cells. A. The principle of FLIM-FRET assay with GFP_comp_ and mCherry as the FRET donor and acceptor, respectively, is schematically drawn; a representative fluorescence decay dataset fitted curve (in red) and residuals for a single pixel within the nucleus of a cell infected with the WSN-PB2-GFP11 virus are shown. B. Fluorescence intensity (left and middle panels) and mean GFP_comp_ fluorescence lifetime (right panels) images of the infected and/or transfected cells. Graphs to the right of the micrographs show the distributions of mean GFP_comp_ fluorescence lifetime values (occurrence of pixels with a given mean lifetime) in the nuclei pointed by yellow arrowheads in GFP_comp_ intensity images (middle right panels). Sketches on the far right show the “observable”, fluorescently labeled species for each sample; virus ideographs indicate viral infection, either with WSN-wt (in positive and negative controls) or with WSN-PB2-GFP11 (in other infected samples).

In our FLIM-FRET measurements, GFP_comp_ was the donor and mCherry was the acceptor (Fig 6A). The mCherry-GFP_comp_ fusion protein, in which the donor and the acceptor are hold in proximity by a 17-amino acid linker, was used as a positive control; however, a “100% FRET efficiency” was not expected for the latter construct since the distance and orientation of the two fluorophores might not be optimal for FRET. Co-expressed non-interacting constructs MBD-GFP_comp_ and mCherry served as the FRET negative control, as the level of expression of mCherry in these samples was higher than or similar to the level of expression of NP-mCherry in experimental samples. We observed that the fluorescence lifetime of GFP_comp_ in the negative control samples does not depend on the presence of the acceptor (compare the mCherry-positive and mCherry-negative cells on Fig 6B, upper panel), indicating that accidental collisions between labeled molecules do not affect FLIM-FRET readout.

In the nucleus of infected cells at 5–7 hpi, the observed FRET efficiency was similar to the value for the positive control (Fig 6B and Table 3, sample #2 compared to sample #1), indicating the presence of species in which PB2-GFP_comp_ and NP-mCherry are in close proximity. These species could correspond to PB2-GFP_comp_ associated with isolated NP-mCherry or with NP-mCherry-containing vRNPs. However, a decrease of PB2-GFP_comp_ fluorescence lifetime indicating substantial FRET was also observed i) when PB2-GFP_comp_ and NP-mCherry were co-expressed in non-infected cells (Table 3, sample #4), and ii) when PB2-GFP_comp_ was co-expressed with the non-oligomerizing NP_R416A-mCherry mutant, in infected cells as well as in non-infected cells (Fig 6B and Table 3, samples #3 and #5). These data are strongly in favor of direct interactions between PB2-GFP_comp_ and free NP-mCherry taking place in the context of infected cells.

**Table 3:** FRET efficiency between PB2-GFP_comp_ and NP-mCherry in the nuclei of HEK-293T cells.

Sample #	Experimental conditions^a^	FRET efficiency^b^
**1**	**mCherry-GFP11 (positive control)**	0.140±0.032**
**2**	**NP-mCherry + WSN-PB2-GFP11 virus**	0.130±0.071**
**3**	**NP_R416A-mCherry + WSN-PB2-GFP11 virus**	0.110±0.069**
**4**	**NP-mCherry + PB2-GFP11**	0.064±0.047**
**5**	**NP_R416-mCherry + PB2-GFP11**	0.100±0.070***

^a^GFP1-10 was transiently expressed in all samples, in addition to the plasmids mentioned in the first column; significant difference with negative control:

** P<0.01;

*** P<0.05

^b^FRET efficiency was calculated on the basis of GFP_comp_ fluorescence mean lifetime values, using the mean lifetime of the negative control as *τ*_*D*_, as described in Materials and Methods (Equation 1). 19 to 22 cells per condition were used, from at least two independent transfection-infection experiments.

## Discussion

To independently visualize the PB2 influenza polymerase subunit and vRNPs in the infected cells, here we combined labeling of PB2 with split-GFP by using the recombinant WSN-PB2-GFP11 virus [33] and labeling of virus-encoded RNPs by spontaneous incorporation of transiently expressed NP-mCherry fusion protein. The latter enabled us to bypass the problem of attenuation of recombinant influenza virus when fluorescent protein tags are fused to the NP. We show that NP-mCherry was incorporated into vRNPs and did not perturb viral replication. An alternative strategy to label vRNPs in live cells, *i*.*e*. transfection of a fluorescently labeled monoclonal antibody, was used by others to characterize quantitatively the intra-cytoplasmic movements of vRNPs containing particles [28]. However, this labeling strategy has fundamental limitations including possible off-target antibody binding and restricted penetration of labeled antibody to certain subcellular compartments. While our manuscript was in preparation the use of a GFP-NP construct to study cytoplasmic trafficking of vRNPs was reported [49]. Here, unlike in the above-mentioned work, the viral NP and PB2 proteins were simultaneously labeled, the incorporation of the labeled species into vRNPs was carefully assessed (Figs 3 and 4), and the mobility and interactions of the NP and PB2 proteins were characterized in the nuclei of infected cells rather than in the cytoplasm.

In our experiments, NP-mCherry-containing species, *i*.*e*. free NP-mCherry, NP-mCherry bound to cellular structure(s) or viral proteins, and NP-mCherry incorporated into either free vRNPs or vRNPs interacting with cellular structures, may exist in various proportions. The same stands true for PB2-GFP_comp_-containing species. Despite of this potential diversity, we could determine the translational diffusion times of two NP-mCherry- and two PB2-GFP_comp_-containing species in infected cells: one showing the same mobility as the monomeric non interacting mCherry and MBD-GFP controls, respectively, and a “slow” one. Comparison of the diffusion times and proportions corresponding to the “slow species” in uninfected *versus* infected cells on one hand, and in infected cells expressing NP-mCherry *versus* NP_R416A-mCherry on the other hand, indicates that “slow species” of NP-mCherry and PB2_comp_ can appear in a non-infectious context and independently of the capacity of NP to oligomerize, but they show a decrease in mobility in infected cells compared to uninfected cells. Thus, our data strongly suggest that not only vRNPs or hetero-trimeric polymerases, but also isolated NP and PB2 proteins can associate to cellular structures [40]. Interestingly, our data also indicate that a significant proportion of NP-mCherry and PB2_comp_ (about 50% and 70%, respectively) remains in a “free-like” state in the nuclei of infected cells. A pool of RNP-free NP and PB2 proteins in the nucleus of infected cells could possibly have a secondary function distinct from transcription/replication of the viral genome.

The combination of two labeling approaches enabled us to detect, by FLIM-FRET, a direct interaction between NP and PB2 in the nucleus of live cells; this interaction, to our knowledge, had only been characterized by biochemical techniques so far [16, 18, 50, 51]. The PB2-NP interaction is of significant interest as the question whether it could substantially affect the range of permissive hosts for a given viral strain remains controversial. There are conflicting reports about whether or not one of the most important mutations responsible for the adaptation of avian influenza viruses strains to human, PB2-E627K, stabilizes the vRNP and contributes to enhanced polymerase activity by facilitating the interaction between PB2 and NP [16–20]. Non-invasive and reliable methods to detect the PB2-NP interaction in live infected cells, such as the combination of NP and PB2 labeling described in the present paper, should help addressing this question. In addition, this method could be adapted to the detection of other protein-protein interactions that occur during infection.

Our FRET data indicate that direct interactions between RNP-free PB2-GFP_comp_ and RNP-free NP-mCherry can occur in the context of an infected cell. Taken together with our FCS data, they suggest that RNP-free NP-PB2 interactions could occur in the nucleus of infected cells. Such interactions could be required for other functions than the NP-polymerase interaction taking place in the context of a vRNP. Three NP residues (R204, W207 and R208) were shown to be required for the NP-polymerase interaction; they are located in a loop at the top of the head domain of NP without any overlap with the NP sequences needed for RNA binding and oligomerization, and are required for viral RNA synthesis [52]. The RNP-free PB2-NP interactions revealed by our FRET data could involve other residues. Combined with mutagenesis and reverse genetics, the simultaneous labeling of NP and PB2 should be of great use for systematic live cell-based studies of these RNP-free NP-PB2 interactions in the context of human and avian host cells.

Multicolor single molecule fluorescence in situ hybridization (smFISH) analysis has recently been used to document where and when vRNPs corresponding to different segments associate with each other [34, 53]. Intermediate assemblies of vRNPs were detected in the cytoplasm, away from virion assembly sites at the plasma membrane, but it remains unclear whether vRNPs are exported from the nucleus individually or as intermediate assemblies. Molecular brightness data from our FCS experiments did not reveal a significant population of complexes containing multiple PB2 proteins in the nucleus, suggesting that multi-vRNP complexes are absent from the nucleus, or are present at a very low level compared to single vRNPs and free PB2 proteins. The labeling approach reported here could also be applied to the tracking of vRNPs in the cytoplasm. It could in principle be adapted to correlative microscopy, which enables one to analyze the same sample by photonic imaging and by electron microscopy (EM), thus combining temporal resolution and molecular specificity with nanometer scale structural information.

In conclusion, application of photonic imaging based on carefully designed labeling, in combination with other techniques, would improve understanding of influenza virus life cycle in the cell. More broadly, our study illustrates the potential of photonic imaging based on carefully designed labeling for virology studies, and provides an example of the characterization of time-post-infection dependent localisation, mobility and interactions of viral proteins and their complexes in nearly-native conditions in live infected cell.

## Supporting Information

S1 Fig Principle of fluorescent tagging of influenza virus PB2 protein using the GFP1-10 –GFP11 complementation system. (TIF)

S2 Fig Effect of Rab11 knock-down on NP immunostaining pattern in A549 cells infected with the WSN influenza virus, as visualized at 19 hpi by immunostaining with the mouse NP 3/1 antibody. Single confocal slices are shown. Scale bar: 10 μm. Yellow boxes highlights the areas for which higher magnification images are shown.
(TIF)

S3 Fig Control of transfection efficiency for evaluation of the effect of NP-mCherry and NP_R416A-mCherry on influenza virus replication. Live HEK-293T cells were transfected with indicated mCherry-containing or control constructs and imaged by fluorescence microscopy at indicated times. The cells were infected with the WSN-wt virus at low MOI at 24 hours post-transfection and the supernatants collected at 72 hpi were titrated by plaque assay (Table 1). A wide-field microscope was used with standard filters for red fluorescence. Scale bar: 100 μm.
(TIF)

S1 Movie A Vero cell transfected with GFP1-10 and infected with the WSN-PB2-GFP11 virus was observed at various times post-infection, as indicated. Green: PB2-GFP_comp_; red, NP-mCherry. Individual color channels and merged images. Time post-infection is indicated. Scale bar: 10 μm; single optical slices; signals in the two color channels were acquired sequentially on a Nipkow spinning disk microscope.
(AVI)
