# Supplementary figures and images for: Time-Resolved Visualisation of Nearly-Native Influenza A Virus Progeny Ribonucleoproteins and Their Individual Components in Live Infected Cells
Source: PLoS One. 2016 Mar 15;11(3):e0149986. doi: 10.1371/journal.pone.0149986 (PMC4792379; doi:10.1371/journal.pone.0149986)

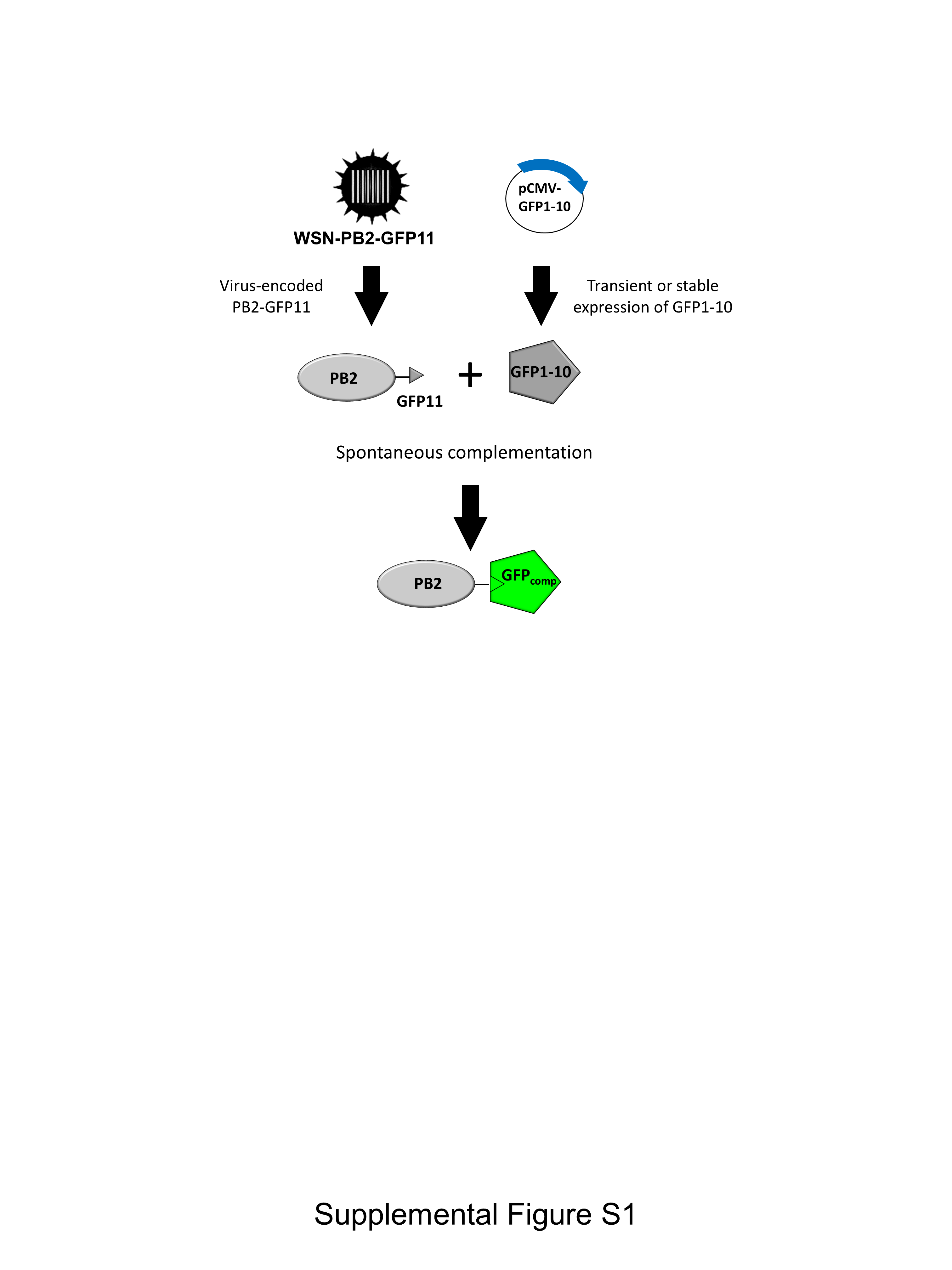

Supplement: S1 Fig — (TIF) [file pone.0149986.s001.TIF]

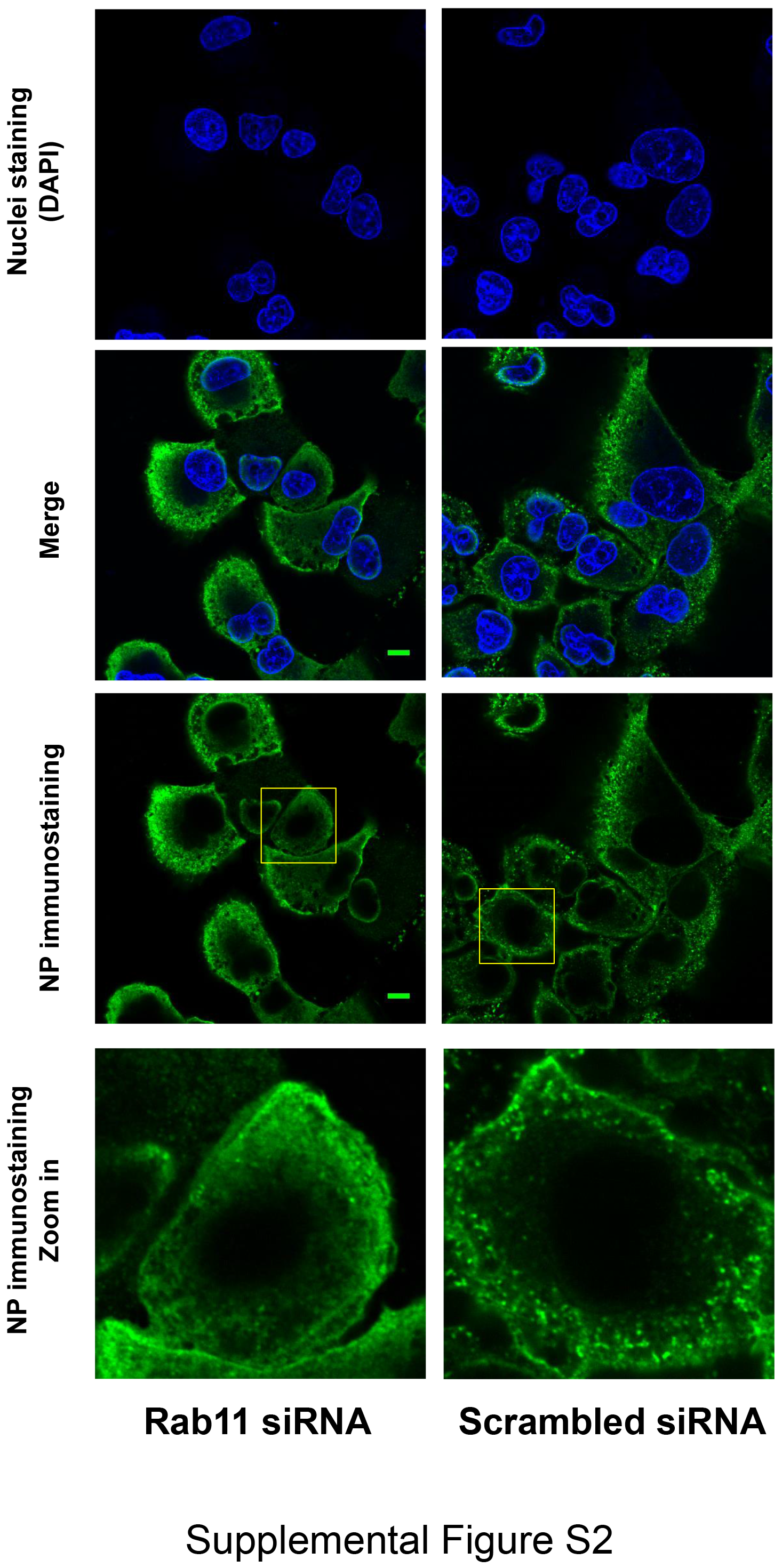

Supplement: S2 Fig — Single confocal slices are shown. Scale bar: 10 μm. Yellow boxes highlights the areas for which higher magnification images are shown. (TIF) [file pone.0149986.s002.tif]

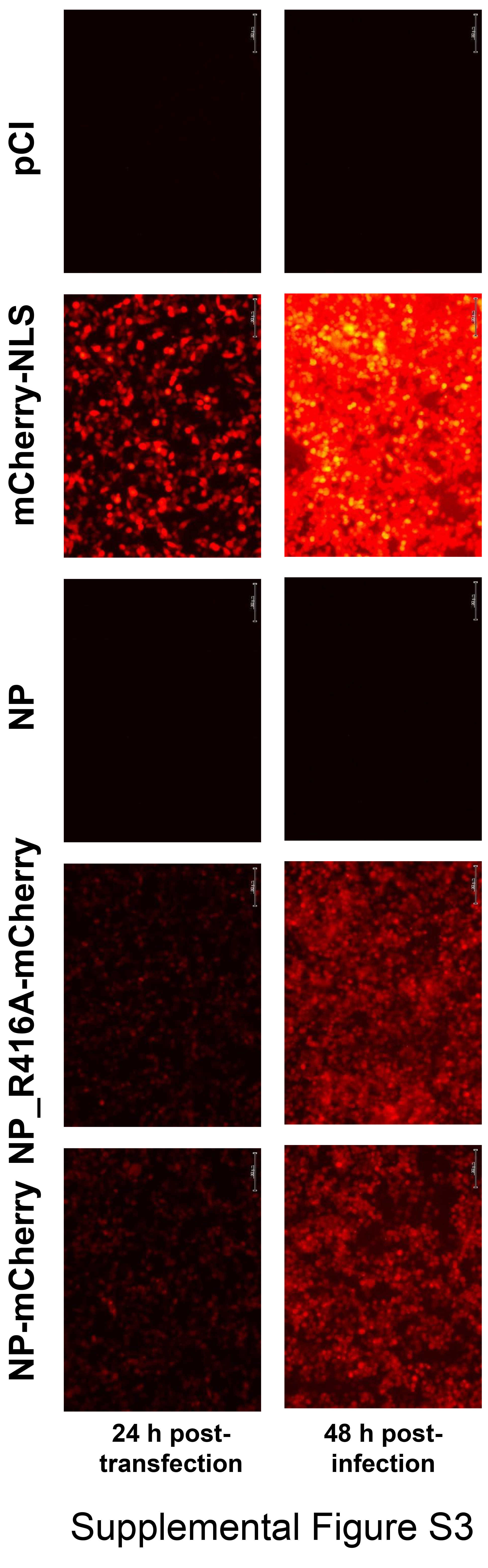

Supplement: S3 Fig — Live HEK-293T cells were transfected with indicated mCherry-containing or control constructs and imaged by fluorescence microscopy at indicated times. The cells were infected with the WSN-wt virus at low MOI at 24 hours post-transfection and the supernatants collected at 72 hpi were titrated by plaque assay (Table 1). A wide-field microscope was used with standard filters for red fluorescence. Scale bar: 100 μm. (TIF) [file pone.0149986.s003.tif]
